# Genomic Prediction for Abortion in Lactating Holstein Dairy Cows

**DOI:** 10.3390/ani12162079

**Published:** 2022-08-15

**Authors:** Robert Wijma, Daniel J. Weigel, Natascha Vukasinovic, Dianelys Gonzalez-Peña, Shaileen P. McGovern, Brenda C. Fessenden, Anthony K. McNeel, Fernando A. Di Croce

**Affiliations:** Zoetis Inc., 333 Portage Street, Kalamazoo, MI 49007, USA

**Keywords:** genetics, genomics, cow abortion, Holstein, selection index, breeding strategy, prediction, standardized transmitting ability

## Abstract

**Simple Summary:**

Pregnancy losses in dairy cattle result in impaired animal health and welfare, as well as economic losses due to increased culling, reduced milk production, calf losses, and increased reproductive costs, among others. Advances in the last few decades have allowed the dairy industry to make significant progress in reproductive efficiency, but pregnancy losses continue to be an unresolved problem. The origin of abortions can be infectious disease, metabolic disorders, heat stress, and a genetic predisposition among many others. We have developed genomic predictions (Z_Abort) to identify Holstein dairy cows with a greater risk of abortion. This allows dairy producers and veterinarians to select more productive and profitable cows. The objectives of the study were to (1) describe the development of the genomic predictions for cow abortions in lactating Holstein dairy cattle based on producer-recorded data and ssGBLUP methodology and (2) evaluate the efficacy of genomic predictions for cow abortions in commercial herds of US Holstein cows using data from herds that do not contribute phenotypic information to the evaluation. The results of the present study show that the genomic predictions for cow abortion trait (Z_Abort) can effectively predict the risk of abortion of lactating Holstein dairy cows and, hence, allow genetic selection towards healthier and more profitable cows.

**Abstract:**

Abortion in dairy cattle causes great economic losses due to reduced animal health, increase in culling rates, reduction in calf production, and milk yield, among others. Although the etiology of abortions can be of various origins, previous research has shown a genetic component. The objectives of this study were to (1) describe the development of the genomic prediction for cow abortions in lactating Holstein dairy cattle based on producer-recorded data and ssGBLUP methodology and (2) evaluate the efficacy of genomic predictions for cow abortions in commercial herds of US Holstein cows using data from herds that do not contribute phenotypic information to the evaluation. We hypothesized that cows with greater genomic predictions for cow abortions (Z_Abort STA) would have a reduced incidence of abortion. Phenotypic data on abortions, pedigree, and genotypes were collected directly from commercial dairy producers upon obtaining their permission. Abortion was defined as the loss of a confirmed pregnancy after 42 and prior to 260 days of gestation, treated as a binary outcome (0, 1), and analyzed using a threshold model. Data from a different subset of animals were used to test the efficacy of the prediction. The additive genetic variance for the cow abortion trait (Z_Abort) was 0.1235 and heritability was 0.0773. For all animals with genotypes (n = 1,662,251), mean reliability was 42%, and genomic predicted transmitting abilities (gPTAs) ranged from −8.8 to 12.4. Z_Abort had a positive correlation with cow and calf health traits and reproductive traits, and a negative correlation with production traits. Z_Abort effectively identified cows with a greater or lesser risk of abortion (16.6% vs. 11.0% for the worst and best genomics groups, respectively; *p* < 0.0001). The inclusion of cow abortion genomic predictions in a multi-trait selection index would allow dairy producers and consultants to reduce the incidence of abortion and to select high-producing, healthier, and more profitable cows.

## 1. Introduction

Reproductive success is one of the main drivers of profitability in dairy herds. Every day a cow remains non-pregnant beyond the established voluntary waiting period results in reduced profit. Pregnancy loss has a substantial negative impact on reproductive efficiency and, therefore, on-farm profitability [1]. The Committee on Bovine Reproductive Nomenclature [2] defines embryonic loss as any pregnancy that ends prior to day 42 of gestation with pregnancy losses after day 42 considered to be fetal loss or abortion. Using a comprehensive breeding, replacement, and economic model, De Vries et al. [3] estimated the value of a new pregnancy ranged between USD -14 and USD 551, depending on DIM at conception, milk yield, and lactation number. For low-producing cows that also get pregnant late in gestation, the value of the pregnancy was the lowest. In addition, the cost of pregnancy loss was also low among low-producing cows, because many of these cows would be culled, even when pregnant, due to low profitability. In contrast, cows with average and high milk yield that experienced a pregnancy loss represented an economic loss even greater than the value of the pregnancy. This is mainly because of increased days open, the opportunity cost of delaying the next lactation, and increased involuntary culling of profitable animals. The cost of pregnancy loss increased with the milk production level and month of gestation, being as high as USD 1373 for a high-yield primiparous cow that aborts at month 7 of gestation. Moreover, this study assumed that once aborted cows would behave as non-pregnant cows; thus, it can be speculated that the real cost of abortion would be even greater. The actual value of pregnancy and pregnancy loss changes with market values of culled cows, heifers, feed, milk, etc., but most research and simulation models support the concept that abortion reduces profitability [4]. Wijma et al. [5] reported that cows that abort have a greater risk of a new abortion if they conceive again, a reduced risk of pregnancy by 400 DIM, and a greater risk of exiting the herd than non-aborted cows. Furthermore, abortions can cause further health problems, such as retained placenta, metritis, endometritis, and pyometra, with the consequent impact on reproduction, milk production, and productive life [6,7,8,9]. Santos et al. [1] estimated that 60% of pregnancies are lost from conception to term. Pregnancy losses in high-producing dairy cows range from 5.4 to 12.8% between the first and second month of gestation [10,11,12], and from 1 to 3.3% during the third month of pregnancy [13,14,15]. Although there are not many reports of abortion rates from 90 days to term, it was estimated at 3–5% by Hovingh [16]. Diagnosis of the etiological cause of abortion represents a real challenge for producers and veterinarians. Among the possible causes of abortion are genetic abnormalities, heat stress, toxic agents, bacteria, viruses, and protozoa, among others [16]. Close to 50% of the fetuses submitted to diagnostic labs yield a confirmed diagnosis, but that means that the other 50% usually remain undiagnosed [1]. Management strategies, such as vaccination, biosecurity measures, use of mycotoxin sequestering agents, and heat stress mitigation, among others, help to reduce but not eliminate the incidence of abortions.

In the last few decades, advances in the understanding of reproductive physiology, the development of new reproductive management strategies, and genetic selection have allowed the dairy industry to drastically reverse a negative trend and increase pregnancy rates [17]. Despite this, pregnancy losses continue to be a problem for which no clear solution has been developed. In this regard, there is an established and ongoing effort by research groups to quantify the genetic component of abortion in cattle. Several recessive haplotypes and mutations that cause embryo or fetal loss, as well as stillbirth, have been identified in the genome of dairy cattle [18,19,20]. The discovery of these haplotypes allows for selective breeding of animals that carry these mutations to avoid homozygotic fetuses (and, thus, prevent pregnancy losses) and reduce the prevalence of these haplotypes in future generations [20]. Precision mating based on genomic data to control these losses can have an annual impact of USD 11,000,000 on the USA dairy industry [21]. Sigdel et al. [22] identified the presence of genomic markers associated with pregnancy loss in dairy heifers and lactating dairy cows. Moreover, Gershoni et al. [23] identified candidate markers to predict the risk of early abortion in Israeli Holstein cattle. This growing evidence that abortion incidence has a genetic component supports the fact that it can be controlled, at least in part, through genetic selection.

Considering the economic impact of pregnancy losses discussed in the previous paragraphs, the development of a multi-trait selection index that includes abortion risk would help select and breed more profitable cows. Selection indices allow dairy breeders and producers to select animals based on a combination of different traits, each of which receives a relative weight. Selection indices provide a way to combine information about many traits into a single number that can rank animals and inform breeding decisions. Initially, selection indices were built accounting only for production traits, hence indirectly selecting against fertility and health traits [24,25] due to the negative correlation between the traits. In the last few decades, the dairy industry has become more conscious that boosting the fertility and health of dairy cows is paramount to maximizing profitability, reducing antibiotic use, assuring animal welfare, and complying with sustainability standards. As a result, modern selection indices include not only production traits but also fitness traits. Attending to these needs of dairy producers and the dairy industry, Zoetis developed the Dairy Wellness Profit^TM^ (DWP$) index in 2016. DWP$ is an economic multi-trait selection index that was formulated to estimate the potential lifetime profitability an animal would generate under dairy economic conditions in the United States and includes cow and calf wellness, production, fertility, functional type, longevity, livability, calving ability, and milk quality traits [26,27]. This index has been validated in commercial dairy farms to select more profitable and healthier cows [27]. In 2020, DWP$ was updated to include additional traits shown to impact the lifetime profitability of a dairy animal; one of those traits was cow abortion.

The objectives of this study were to (1) describe the development of the genomic predictions for cow abortions in Holstein dairy cattle based on producer-recorded data and ssGBLUP methodology and (2) evaluate the efficacy of genomic predictions for cow abortions in commercial herds of US Holstein cows using data from herds that do not contribute phenotypic information to the evaluation. In the current study, we hypothesized that cows with the highest genetic risk for abortion would have a higher phenotypic incidence of abortion.

## 2. Materials and Methods

### 2.1. Data Sources for Genetic Evaluation

Data from 1991 to date were available from 279 herds from 24 states in the USA with an average of 13,759 phenotypic records per herd. Signed consents were obtained from herd owners, and herds were not monitored or economically compensated by Zoetis. Herd performance and pedigree information was retrieved from the on-farm herd management software.

The methodology used in this study was similar to that reported in McGovern et al. [28]. The Zoetis Genotyping Laboratory (Zoetis Genetics, Kalamazoo, MI, USA) provided the genotypes with a wide range of SNPs from low (3000 to over 35,000 SNPs) to medium (50,000–80,000 SNPs) density, and any genotype with <40,000 SNPs were imputed using the program FImpute [29] to 45,245 SNPs used in the genomic evaluation.

### 2.2. Data Editing and Trait Definition for Genetic Evaluation

Similar to McGovern et al. [28] and described by Norman et al. [30], data editing included animal identification verification for accuracy and consistency across data files. A lactation record with a valid calving date and a lactation number, a calving interval between 250 and 999 days, and appropriate age at each calving were required for including an animal in the analysis. Animals recorded as males in the pedigree or having a calving date preceding their birth date were removed.

The trait for cow abortion (**Z_Abort**) was defined as the loss of pregnancy between 42 and 260 days of gestation. Animals having recorded abortions before 42 days of pregnancy were removed from the data. Abortions recorded after 260 days of gestation were considered full-term pregnancies and the animals were defined as healthy. To be considered as aborted, a cow must have had a breeding event followed by a positive pregnancy diagnosis and a recorded abortion event. Healthy animals must have been confirmed pregnant and have reached at least 260 days of gestation without an abortion event. Animals sold or died before having an opportunity to reach 260 days of pregnancy were removed. Abortion was treated as a binary event (abort = 1, healthy = 0). Only one abortion event per lactation was considered. Further, each animal was required to have a known service sire, as well as an estimated 305 days ME milk yield and days open within biological limits.

Contemporary groups (HYS) were formed by combining herd, year, and season of calving, considering 4 seasons within each calving year: winter (December–February), spring (March–May), summer (June–August), and Fall (September–November). If within a herd by year and season of calving (HYS) group there were not at least twenty records and at least one recorded abortion event the group was omitted from the analysis because it was assumed that the herd did not record abortions at all or did not record them during that time period.

### 2.3. Statistical Model for Genetic Evaluation

The analyses for the present study were conducted largely as described by Gonzalez-Peña et al. [31]; the following threshold animal model with repeated observations was used to conduct the analysis:

λ = *Xβ* + *Z_h_h* + *Z_s_s* + *Z_a_a* + *Z_p_p* + *e* (1)

where λ represents a vector of unobserved liabilities to abortion; *β* is the vector of fixed effects, with the corresponding incidence matrix *X*; fixed effects included parity (1, 2, 3, 4, and ≥5), breed composition of the embryo (1—purebred, 2—crossbred), and the linear covariates of milk yield and days open; *h* is the random herd–year–season effect, where *h* ~ *N*(0, *Iσ_h_*^2^), with the variance *σ_h_*^2^; *s* is the random effect of the service sire, with *s* ~ *N*(0, *Iσ*_*s*_^2^); *a* is the random animal effect, with *a* ~ *N*(0, *Hσ_a_*^2^), where *σ_a_*^2^ is the additive genetic variance and *H* is the pedigree relationship matrix augmented using genotypes; *p* is the random effect of the permanent environment with *p* ~ *N*(0, *Iσ_p_*^2^), where *σ_p_*^2^ is the permanent environment variance, and e is the residual, where *e* ~ *N*(0, *I*). *Z_h_*, *Z_a_*, and *Z_p_* are incidence matrices corresponding to the random effects of herd–year–season, animal, and permanent environment, respectively; and I is the identity matrix.

Variance components were estimated using the program THRGIBBS1F90 version 2.108 from the BLUPF90 family [29] without genotypes. The genomic breeding values were obtained using the programs from the BLUPF90 family [30] with a univariate threshold model based on a single-step genomic BLUP methodology (ssGBLUP). The inverse of the traditional pedigree relationship matrix, *A*^−1^, was replaced by the inverse of the *H* matrix that combines pedigree and genomic relationships [31,32]:

(2) $$H^{-1}=A^{-1}+\left[\begin{array}{cc} 0 & 0\\ 0 & G^{-1}-A_{22}^{-1} \end{array}\right]$$

where *A*^−1^ is an inverse of the pedigree relationship matrix, *G*^−1^ is an inverse of the genomic relationship matrix and *A*_22_^−1^ is an inverse of the pedigree–relationship matrix for genotyped animals only. The ‘algorithm for proven and young animals’ (APY) was applied [33]. The program CBLUP90IOD2 version 3.39 with a core of 25,000 randomly selected animals was used to obtain genomic breeding values using a preconditioned conjugate gradient (PCG) with the number of rounds set to 200. The genomic matrix conditioning parameters tau and omega were set to 1.0. Inbreeding was considered when constructing the pedigree relationship matrix. The reliabilities of estimated breeding values (EBVs) were obtained with the program ACCF90GS version 2.54 (see McGovern et al. [28] for details).

The transformation from raw EBVs (solutions from the CBLUP90IOD2 program) to predicted transmitting abilities (PTAs) was described in detail by McGovern et al. [28]. Briefly, the probability that a standard normal variable with the mean equal to this solution and the variance of one exceeds the threshold was calculated for each animal, then multiply by 100, divided by 2, and then deviated from a base population. For ease of interpretation, gPTAs were transformed into standardized transmitting abilities (STAs), with a mean of 100, a standard deviation of 5, and the reversed sign (so that higher values represent a lower risk of abortion) as per McNeel et al. [25]. Correlations between Z_Abort STAs and other Zoetis Wellness trait STAs, and trait (g)PTAs in the US national genetic evaluation (Council of Dairy Cattle Breeding) were estimated using product–moment (Pearson) correlations, similar to Gonzalez-Peña et al. [31].

### 2.4. Inclusion of Abort Prediction in a Multi-Trait Selection Index

For the purposes of this study, (1) the Z_Abort STA, its phenotypic correlations, and genetic relationships with other traits were estimated, (2) the economic value of the Z_Abort trait as it relates to its contributions to a dairy animal’s lifetime profitability was estimated (by determining the economic value of all incomes and losses for a 1-unit increase in the abortion trait), and (3) the STA for Z_Abort was multiplied by its corresponding economic weight, alongside all other traits in the lifetime merit selection index (DWP$ index), which were then summed together to determine an animal’s overall selection index value [27,34].

### 2.5. Demonstration of Evaluation Efficacy

The efficacy of Z_Abort predictions was established using a subset of animals that did not contribute phenotypes to the genetic evaluation. Data from 6922 females (14,068 observations) belonging to 5 commercial herds from the USA with good record-keeping practices for abortion events (herd abortion incidence similar to the observed average in the genetic evaluation population and industry standards) were included. 

Importantly, Z_Abort STA predictions for the selected animals (lactations 1 to 4) were generated from phenotypic data available on 31 December 2012 to ensure that an animal’s own performance data were not contributing to the genetic evaluation and STAs would, in effect, be those of a heifer. Genomic predictions for cow abortion were used to rank animals within herd and birth year and season and assign them to quartiles based on their Z_Abort STA (genetic groups: worst 25%, 26–50%, 51–75%, and best 25%) similar to what was reported in McGovern et al. [28]. As a side analysis, the same approach was used to generate Daughter Pregnancy Rate (DPR) genomic groups. Statistical analyses were performed using PROC GLIMMIX form SAS (version 9.4, SAS Institute Inc, Cary, NC, USA) with binomial distribution and logit link function including genetic prediction quartile, lactation (1–4), milk production (305ME), season of conception, type of semen used for breeding (sexed sorted, conventional dairy breed, and conventional beef breed) as fixed effects, and herd as a random effect. Results are reported as back-transformed least square means (i.e., abortion incidence) and standard error of the means; differences between groups were considered statically significant if *p* < 0.05. The cost of an abortion was assumed as USD 555 [3]. A second statistical model was used to estimate the predicted probability of abortion by Z_Abort STAs and lactation. For this, Z_Abort STA and lactation (1–4) were included as fixed effects, and the animal was included as a random effect. 

The frequencies of reproductive Holstein haplotype (HH) carriers for AI sires and for cows within each Z_Abort STA quartile were calculated for HH0-HH5. The latter was analyzed using PROC GLIMMIX from SAS (version 9.4, SAS Institute Inc, Cary, NC, USA) with a binomial distribution and logit link function including Z_Abort STA quartile as a fixed effect. The frequency of the mating of two carriers for a given HH was calculated for cows that did and did not abort for cows bred to AI sires with known HH status (i.e., matings with a beef breed or herd bull were eliminated for this analysis). 

## 3. Results

### 3.1. Data Characteristics for Genetic Evaluation

The number of phenotypic records, genotypes, animals in the evaluation, and the incidence of abortion as of April 2022 are described in Table 1.

### 3.2. Variance Components and Summary Statistics for Genetic Evaluation

Variance components estimations for cow abortion in Holsteins are shown in Table 2.

**Figure 1:**
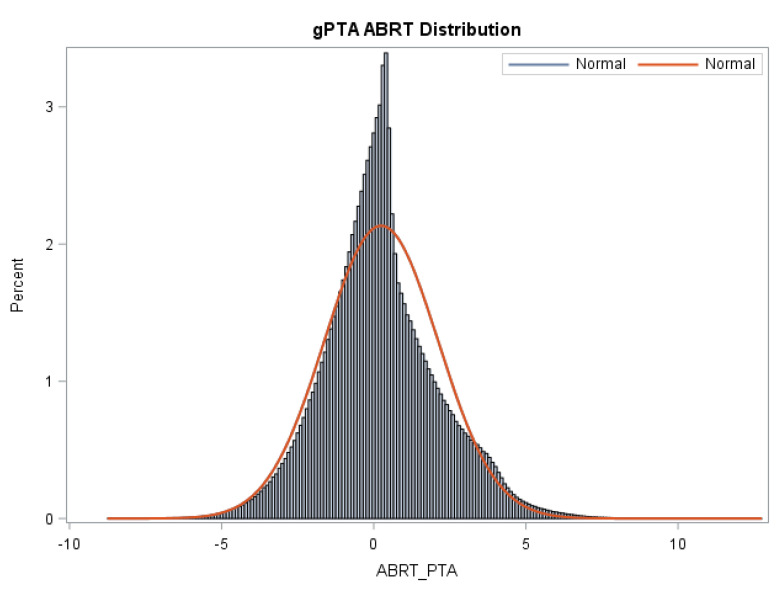
Distribution of gPTAs (Z_Abort PTA) for abortion for all animals in the Holstein evaluation. An appreciable variation in gPTAs is observed in the Holstein population sampled in the present study.

The means, standard deviations, and reliabilities of gPTAs, STAs, and reliabilities for Z_Abort are presented in Table 3. Figure 1 shows the distribution of gPTAs for abortion.

### 3.3. Correlations of Abortion with Other Traits

The product–moment (Pearson) correlations of genomic predictions for cow abortion (Z_Abort STA) with predictions for the lifetime merit selection index (DWP$ index) and other Zoetis wellness and fertility traits, and the correlations of Z_Abort with economically important traits and indexes in the national genetic evaluation produced by the Council on Dairy Cattle Breeding (CDCB) are presented in Table 4.

### 3.4. Demonstration of Evaluation Efficacy

**Figure 2:**
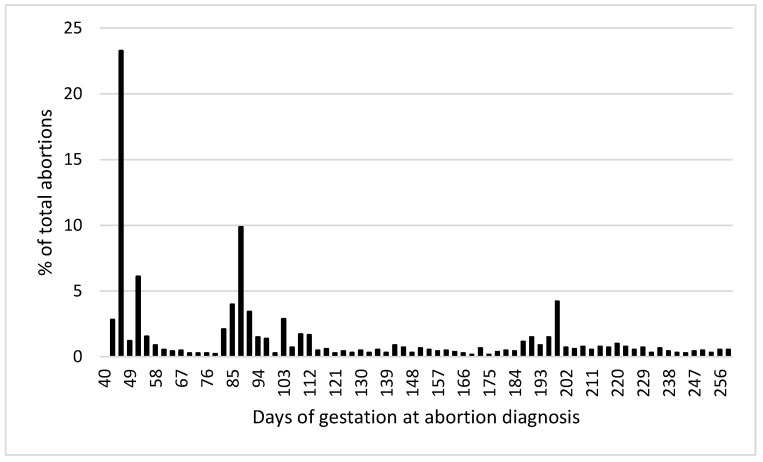
Distribution of abortion incidence by days of gestation at the time of abortion diagnosis.

A total of 6922 cows and 14,068 pregnancies were included in the analysis. The overall incidence of abortion was 12.8% (1800/14,068). Figure 2 shows the distribution of abortion incidence by days of gestation at the time of abortion diagnosis. There was no significant effect (*p* > 0.1) of milk production (305ME), the season of conception, type of semen used for breeding (sexed sorted, conventional dairy breed, and conventional beef breed), or cow abortion group by lactation interaction on abortion incidence. Therefore, these effects were removed from the final model.

#### 3.4.1. Cow Abortion for the Genetic Groups

The observed results demonstrated the association between the genetic prediction of cow abortion and phenotypic incidence of abortion. Differences in observed cow abortion (marginal means) were statistically significant between Z_Abort genetic groups (*p* < 0.0001). As shown in Table 5, abortion incidence in the best Z_Abort group was 34% (5.6 percentage points) lower than in the worst group, and the difference in the economic cost was estimated as an average of USD 31 per cow. The worst genomic cow abortion (Z_Abort) group had the greatest (*p* < 0.05) abortion incidence, followed by the next quartile, and the best two groups were not statistically different. There was an effect of the lactation group (*p* = 0.0001; Table 6) on abortion incidence because it was greater (*p* < 0.05) for the third and fourth than for first and second lactation cows. To better understand the added value of Z_Abort to the currently available fertility traits, we explored if there was any existing relationship between DPR gPTA and abortion incidence. We observed a significant effect of DPR gPTA quartile on abortion incidence (*p* = 0.0003; Table 7), with a difference of 23% (3.5 percentage points) between the best and the worst quartile and an estimated difference in the economic cost of USD 20 per cow. The worst genetic group had the greatest abortion incidence (*p* < 0.05), but the best genetic group was not different from the second worst group.

**Figure 3:**
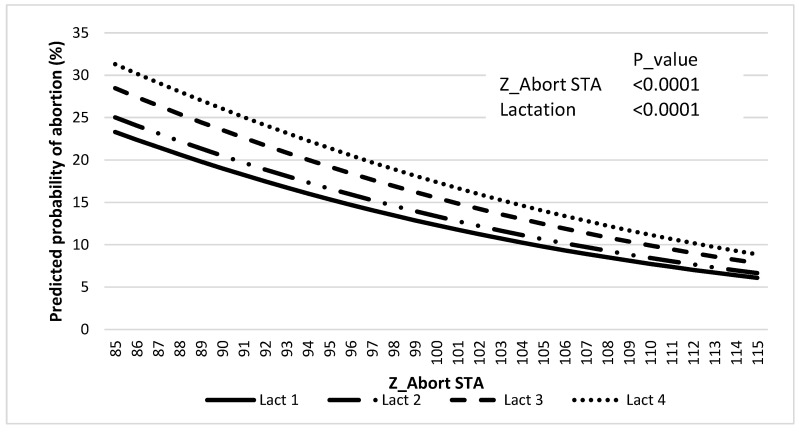
Association between predicted probability of abortion and Z_Abort STAs for lactations 1 to 4.

There was an effect of Z_Abort STA (*p* < 0.0001) and lactation (*p* < 0.0001) on the predicted probability of abortion, where the latter increased as Z_Abort decreased (Figure 3, and Table 6).

#### 3.4.2. Holstein Haplotypes Frequencies

In order to explore any possible bias in the current evaluation due to the prevalence of reproductive Holstein haplotype(s) (HH), we performed an analysis of the proportion of cows within each Z_Abort genetic group and AI sires that were carriers for HH0, HH1, HH2, HH3, HH4, and HH5. Results for HH6 were not available for cows at the time of the evaluation but no AI sire mates were carriers of this defect. Twenty-four cows were eliminated from this analysis because their HH results were not available, and their service sires were recorded as herd bulls. The frequencies of HH carriers for sires and the matings of carriers for cows that aborted and cows that did not abort are reported in Table 8. Table 9 shows the frequencies of HH carriers for cows in the different Z_Abort STA quartiles. The frequency of HH2 was affected by Z_Abort quartile (*p* < 0.001) and was lesser (*p* < 0.05) in the best genetic group. The frequency of HH3 and HH4 tended to be affected by Z_Abort genetic group (*p* = 0.05 and *p* = 0.07, respectively.

## 4. Discussion

Although genetic and genomic associations with abortion in cattle have been previously described in the literature [22,23,35,36], to the best of our knowledge this is the first report to describe abortion risk in a genetic evaluation and demonstrate efficacy to predict actual abortion events using real commercial herd data, and subsequently include it in a lifetime merit selection index.

The additive genomic variance observed in this study was greater than previously reported by others for abortion and/or embryo loss [23,35,36], which explained more than 12% of the variability in abortion incidence. The heritability estimates for abortion were similar to those reported in previous studies [22,23,35,37], although they were lower than the estimates reported by Bamber et al. [38] and Sigdel et al. [22] (multiparous cows only). Even though these studies, including this one, have used different approaches to evaluate genomic markers of abortion risk, the fact that heritability and additive genetic variance are greater than 0 supports the hypothesis that it is feasible to select toward reduced abortion risk based on genomic evaluations.

Incidences of abortion (42 to 260 days after AI) in the genetic evaluation (11.7%) and the validation population (12.8%) were similar to previous reports [1]. Abortion incidence distribution across gestation periods for the validation data set was also in accordance with what has been previously described by others, with greater incidence in the first trimester (i.e., 58%). The incidence of abortion increased with parity within the validation population, similar to what was reported by Keshavarzi et al. [39], but different from what was observed by others [40]. Regardless, results presented in Figure 3 clearly indicate that for lactations 1 to 4, the predicted probability of abortion increases with lower Z_Abort STA values. It can be appreciated that the magnitude of this effect is greater for more mature cows. This may have an important economic significance because mature cows that get pregnant early in lactation (and maintain that pregnancy to term) are more profitable [41], and abortions of those tend to represent the greatest economic cost [3].

The influences of the HH defects in this study were minimal, with only four abortions involving mates of carriers. The low frequency of HH carriers among the genomically confirmed AI service sires, the lack of difference in the proportion of carriers among cows that did or did not abort, and the similarity in the frequency of carrier animals through Z_Abort quartiles for most HH allow us to assume that the abortion incidence was not influenced by the matings of HH carriers. Despite this, HH2 and HH4 were (or tended to be) less frequent in the best quartile for Z_Abort STA. This is not surprising because although HH need to be in a homozygous state to be lethal, the possibility that these markers are also related to some genes with an additive effect cannot be ruled out. Furthermore, we cannot disregard the possible existence of other unknown HH that could be associated with Z_Abort predictions.

Genomic STA for Z_Abort showed a positive correlation with other wellness traits related to reproductive efficiency and animal health. The positive genetic correlation between reduced abortion risk and reduced risk of twin gestation has been previously described by our group [28], and a phenotypic correlation between these two traits has been described in several reports [42,43,44]. Moreover, we observed a positive genetic correlation between Z_Abort and reduced risk of retained placenta and metritis, which is in accordance with phenotypic observations where aborted cows had a greater probability of suffering retained placenta [6,8] and metritis [7,9]. Furthermore, in the present study, we observed that aborted cows had an increased incidence of retained placenta (3.0 vs. 1.2% for aborted and not aborted cows, respectively) and metritis (7.6 vs. 4.6% for aborted and not aborted cows, respectively). These observations, in addition to a positive genetic correlation with a genomic prediction for cow livability, suggest that selecting cows with reduced abortion risks would also contribute to a reduction in the incidence of other health events that directly impact reproduction, cow livability, and profitability.

Interestingly, although previous studies have shown an increase in pregnancy loss in cows suffering from mastitis [39,45] and we have observed a similar trend (15.1 vs. 11.2% for cows with and without a mastitis event, respectively), the observed correlation between Z_Abort and Z_Mastitis is weak. An explanation for this can be that most of the studies reviewed by Dahl et al. [45] were focused on the pre-fetal period (i.e., the first 45 days of pregnancy) whereas we only evaluated those pregnancy losses that occurred beyond day 42 of gestation. The positive correlation with Z_Respiratory might be explained by increased resistance to respiratory viruses, which are usually associated with infectious abortions. This holds for Z_Calf Respiratory, although in a smaller magnitude. Interestingly, there was a positive genetic correlation between Z_Abort and Z_Calf Livability. Previous studies have suggested that markers for abortion are related to genes governing the development of the placenta and fetal immune system, among other functions [22,37]. Therefore, it is possible that cows with high risks of abortions will give birth to calves that have compromised in utero development and with a greater risk of early death or disease. This may have an important implication for the selection of recipients for high-value embryos, because, besides increasing the probability of the gestation to term, it would increase the probability of survival for the calf.

The positive genetic correlation of Z_Abort with DPR and CCR gPTA was relatively low, but it should be noted that the correlations are not adjusted for the reliability of the predictions (42% for Z_Abort). The magnitude of the correlation between DPR gPTA and Z_Abort STA and the observation that the maximum overlap between quartiles is 8% (Table 10) suggest that these two traits are predicting the risk of abortion through different mechanisms. This confirms that Z_Abort adds important and valuable information to abortion risk prediction and selection for more fertile, healthier, and profitable cows. The negligible magnitude of the correlation with HCR is within expectations because only lactating cows were included in the current evaluation.

There is strong evidence supporting that high-yield dairy cows have lesser plasma concentrations of progesterone [46], which is associated with decreased embryo quality and impaired embryonic and fetal development. Thus, the negative correlation between Z_Abort and important production traits (milk, fat, and protein) is not surprising. On the other hand, neither we nor other authors who analyzed the effect of milk production on abortion incidence observed a significant effect [39,40]. Considering that most evidence regarding the increase of pregnancy loss with high milk yield points towards a large effect on early embryo mortality and the negative correlation of Z_Abort with production traits, it could be hypothesized that there might be an association between Z_Abort and early embryo loss. It is important to note that, although in the current study the days of pregnancy at the time of abortions were registered as after day 42, in most of the cases it was not possible to determine the exact day of abortion because during the first trimester most abortions are diagnosed at pregnancy reconfirmation (usually after day 50) or because cows are observed in estrus. Therefore, some of these pregnancy losses diagnosed as abortions could actually be embryonic losses. Considering that close to 60% of abortions occurred during the first 90 days of gestation, it can be hypothesized that many of the abortions predicted by Z_Abort were actually embryonic losses. Although this hypothesis requires further research to be confirmed, it is of great scientific and economic significance because the prediction and control of early pregnancy losses are some of the most important challenges in the dairy industry today. 

The negative correlations with production traits might be a drawback for including Z_Abort as a selection trait in a genetic improvement program. Including Z_Abort (5% weight) in the DWP$ index, along with other fertility and health traits, resulted in positive selection towards cows with fewer abortions. Estimated response to selection for DWP$ (updated April 2022) shows that an increase of 1 SD would result in an increase of 219 pounds for Milk gPTA, 20 pounds for Fat gPTA, and 11 pounds of Protein gPTA coupled with an increase of 0.19 in Z_Abort STA (Table A1). Conversely, the negative correlation of Z_Abort STA and NM$ index suggests that the use of this index for selection will continue to increase the incidence of abortions. 

The magnitude of the improvement gained in Z_Abort through DWP$ might seem marginal if only the female selection is considered. Having the abortion trait predictions for sire selection will likely lead to the removal of some outliers with poor genetic merit and the impact could be of greater magnitude. 

Over the last decades, advancements in reproductive management and genetic selection have allowed the dairy industry to reverse a negative trend in reproductive performance and increase pregnancy rates. Despite this, controlling pregnancy loss and abortion are still a pendent assignment. The evaluation presented and validated in the present study provides the industry with a new tool that could greatly impact reproductive efficiency and cow profitability for future generations of dairy cows. To assess the impact of this trait, we performed a simulation analysis using the model described by DeVries [47] in which we compared two 1000 milking cows herds with different abortion incidences for Lact 1 to 4. One herd was assigned an abortion incidence for the worst Z_Abort quartile and the other for the best quartile. The model estimated a USD 17,000 difference in annual profit in favor of the herd with abortion incidence that corresponded to the best 25% for Z_Abort STA. In addition, the model does not account for the increased incidences of metritis, retained placenta, and other health events related to abortion. Thus, it can be assumed that the real difference in profitability would be even greater, providing evidence of the economic impact that reducing abortion risk through genetic selection can have.

## 5. Conclusions

Results from the current study provide additional evidence that on farms, producer-recorded data can be successfully used for performing genetic evaluations in Holstein cows. Furthermore, the results support our hypothesis that the newly developed genomic predictions can effectively identify cows with high, low, and intermediate risks of abortion within a Holstein cattle population. This indicated that animals with higher Z_Abort STA predictions had lower observed abortion values than animals with lower Z_Abort STA predictions. It follows that decisions made in selection, management, and breeding to increase the average Z_Abort STA value within a given herd would be expected to decrease average abortion incidence. Decreasing the incidence of abortion through direct selection on genetic prediction for abortion (Z_Abort) can play an important role in a comprehensive reproductive management strategy for dairy operations. Including the Z_Abort trait in selection indexes and programs, coupled with state-of-the-art reproductive and health management, can help the dairy industry overcome the difficult challenge of reducing pregnancy loss in high-yield dairy cows. 

## Figures and Tables

**Table 1:** Characteristics of the data in the genomic evaluation for abortion in Holsteins (April 2022).

Item	Count
Total animals in the evaluation	4,955,087
Phenotypic records total	3,838,805
Animals with phenotypes	2,038,425
Animals with genotypes	1,662,251
Animals with genotypes and phenotypes	109,267
Incidence of abortion	11.67%

**Table 2:** Estimated variance components for abortion in Holsteins.

Trait	σ^2^_g_	σ^2^_pe_	σ^2^_hys_	σ^2^_ss_	σ^2^_e_	h^2^	r^2^
Z_ABORT	0.1235	0.0941	0.3748	0.0049	1.0	0.0773	0.1362

σ^2^_g_ = genetic additive variance; σ^2^_pe_ = permanent environment variance; σ^2^_hys_ = herd–year–season variances; σ^2^_ss_ = service sire variance; σ^2^_e_ = residual variance; h^2^ = heritability; r^2^ = repeatability.

**Table 3:** Means, standard deviations, minimum and maximum of gPTAs, STAs, and reliabilities for different groups of animals for abortion in Holsteins.

All Animals with Genotypes					
Variables *	N	Mean	Std Dev	Minimum	Maximum
Z_Abort_PTA	1,662,251	−0.01	2.1	−8.8	12.4
Z_Abort _STA	1,662,251	99.0	4.8	71.0	119.0
Z_Abort _REL	1,662,251	42.4	6.0	0.0	99.6
Animals with genotype (no phenotype, no progeny)					
Z_Abort_PTA	1,232,143	0.1	2.1	−8.8	11.8
Z_Abort _STA	1,232,143	98.8	4.7	73.0	119.0
Z_Abort _REL	1,232,143	41.1	5.3	0.0	62.4
Animals with both genotype and phenotype (no progeny)					
Z_Abort_PTA	39,036	−0.5	2.3	−8.1	12.4
Z_Abort _STA	39,036	100.1	5.1	71.0	117.0
Z_Abort _REL	39,036	49.5	4.5	10.1	62.1

* Z_Abort _PTA = gPTAs for abortion; Z_Abort _STA = STAs for Abortion; Z_Abort _REL = reliabilities of gPTAs for abortion.

**Table 4:** Correlation of Z_Abort STA with Zoetis genetic evaluation for wellness traits STAs in Holsteins (n = 1,542,856) and CDCB traits gPTAs.

		Zoetis Wellness Traits *												
	DWP$	Z_CALF DIAR	Z_CALF RESP	Z_CALF LIV	Z_RETP	Z_METR	Z_MAST	Z_LAME	Z_KETO	Z_DA	Z_MFV	Z_TWIN	Z_RESP	Z_CYST
Z_Abort	0.017	0.013	0.078	0.190	0.285	0.189	0.087	0.068	0.037	0.039	0.069	0.284	0.145	0.013
	CDCB Traits **													
	NM$		Milk		Fat		Prot	PL		LIV	SCS	DPR	HCR	CCR
Z_Abort	−0.180		−0.224		−0.254		−0.264	−0.016		0.184	−0.005	0.289	0.051	0.236

* Z_Abort = cow abortion; Z_CALF DIAR = calf scours; Z_CALF RESP = calf respiratory disease; Z_CALF LIV = calf livability; Z_RETP = retained placenta; Z_METR = metritis; Z_MAST = mastitis; Z_LAME = lameness; Z_KETO = ketosis; Z_DA = displaced abomasum; Z_MFV =milk fever; Z_TWIN = twin; Z_RESP = cow respiratory disease; Z_CYST = cystic ovaries. ** NM$ = net merit; Milk = milk yield; Fat = fat yield; Prot = protein yield; PL = productive life; LIV = cow livability; SCS = somatic cell score; DPR = daughter pregnancy rate; HCR = heifer conception rate; CCR = cow conception rate.

**Table 5:** Least squares means for genomic cow abortion (Z_Abort STA) groups (n = 4) abortion incidence (*p* < 0.0001), SEM of the genetic groups, and estimated abortion cost per cow. ^a–c^ Different letters within the same column represent *p* < 0.05.

Z_Abort STA Genetic Group	Marginal Means (Incidence)	SEM	Abortion Cost per Cow ($)
Worst 25%	0.166 ^a^	0.013	92
25–50%	0.136 ^b^	0.011	76
50–75%	0.115 ^c^	0.010	64
Best 25%	0.110 ^c^	0.010	61

**Table 6:** Least squares means for lactation groups (n = 4) abortion incidence (*p* < 0.0001), and SEM. ^a,b^ Different letters within the same column represent *p* < 0.05.

Lactation Group	Marginal Means (Incidence)	SEM
Lact 1	0.112 ^a^	0.009
Lact 2	0.120 ^a^	0.010
Lact 3	0.140 ^b^	0.012
Lact 4	0.154 ^b^	0.016

**Table 7:** Least squares means for DPR gPTA genetic groups (n = 4) abortion incidence (*p* = 0.0003), SEM of the genetic groups, and estimated abortion cost per cow. ^a–c^ Different letters within the same column represent *p* < 0.05.

DPR gPTA Genetic Group	Marginal Means (Incidence)	SEM	Abortion Cost per Cow ($)
Worst 25%	0.151 ^a^	0.013	USD 84
25–50%	0.127 ^bc^	0.011	USD 71
50–75%	0.133 ^b^	0.011	USD 74
Best 25%	0.116 ^c^	0.010	USD 64

**Table 8:** Frequencies of Holstein haplotype (HH) carriers for genomically-tested AI bulls for breeding that resulted in abortion, and for cows that did and did not abort.

	HH0	HH1	HH2	HH3	HH4	HH5
AI Bulls	2.6%	0.4%	1.6%	2.4%	0.2%	4.8%
Abort	4.4%	2.5%	4.7%	6.4%	1.6%	2.8%
No Abort	4.7%	2.6%	3.7%	6.9%	1.1%	3.2%
Carrier × carrier matings (n)	1	1	1	0	0	1

**Table 9:** Frequencies (%) of Holstein haplotype (HH) carriers for Z_Abort STA genetic groups. ^a,b^ Different letters within the same column represent *p* < 0.05.

Z_Abort STA Genetic Group	HH0	HH1	HH2	HH3	HH4	HH5
Worst 25%	4.5	2.7	5.0 ^a^	6.1	0.1	2.5
25–50%	3.9	2.6	4.2 ^a^	5.9	0.1	3.4
50–75%	5.2	3.9	4.1 ^a^	8.7	0.1	3.3
Best 25%	4.2	2.5	2.1 ^b^	6.7	0.04	4.7
*p*-Value	0.19	0.83	<0.001	0.07	0.05	0.24

**Table 10:** Proportion of observations in the validation data set that share the same quartile for Z_Abort STA and DPR gPTA.

		DPR gPTA Genetic Group			
		Worst 25%	25–50%	50–75%	Best 25%
Z_Abort STA Genetic Group	Worst 25%	8.2	6.8	5.3	3.9
	25–50%	6.8	6.8	6.3	5.7
	50–75%	5.4	6.1	7.3	7.2
	Best 25%	4.2	5.6	6.4	8.1

## Data Availability

Data available on request due to restrictions (privacy). The data are not publicly available due to privacy and 3rd parties agreements.

## Appendix A

**Table A1:** Expected response to the selection expressed in units of the underlying trait when the average DWP$ 2022 is increased by 1SD. Adapted from Zoetis data on file, Technical Bulletin CLR-00428, Table 2.

Trait	Expected Response to Selection
**Fat (Kg)**	9.1
**Protein (Kg)**	5
**Milk (Kg)**	99.3
**Productive Life (mo.)**	1.42
**Cow Livability (%)**	0.66
**Somatic Cell Score (log)**	−0.07
**Residual feed Intake**	−5.17
**Body Size Composite (pts)**	−0.21
**Udder Composite (pts)**	0.27
**Feet and Leg Composite (pts)**	0.08
**Daughter Pregnancy Rate (%)**	0.16
**Heifer Conception Rate (%)**	0.31
**Early First Calving**	0.68
**Cow Conception Rate (%)**	0.43
**Calving Ability ($)**	5.10
**Zoetis Mastitis (STA)**	1.54
**Zoetis Metritis (STA)**	1.90
**Zoetis Retained Placenta (STA)**	0.56
**Zoetis Displaced Abomasum (STA)**	1.04
**Zoetis Ketosis (STA)**	1.73
**Zoetis Lameness (STA)**	1.19
**Zoetis Calf Respiratory (STA)**	0.27
**Zoetis Calf Scours (STA)**	−0.07
**Zoetis Calf Livability (STA)**	0.38
**Zoetis Cow Respiratory (STA)**	0.76
**Zoetis Cystic Ovary (STA)**	0.22
**Zoetis Twinning (STA)**	0.61
**Zoetis Cow Abortion (STA)**	0.19
